# Emerging Trends and Hot Spots in Sepsis-Associated Encephalopathy Research From 2001 to 2021: A Bibliometric Analysis

**DOI:** 10.3389/fmed.2022.817351

**Published:** 2022-02-28

**Authors:** Yizhe Zhang, Sifan Chen, Weitian Tian, Hui Zhu, Weiwei Li, Wanbing Dai, Xiao Zhang, Xiyao Gu, Diansan Su

**Affiliations:** Department of Anesthesiology, Renji Hospital, Shanghai Jiao Tong University School of Medicine, Shanghai, China

**Keywords:** sepsis-associated encephalopathy, bibliometric analysis, hot spots, CiteSpace, VOSviewer, co-citation analysis

## Abstract

**Study Objectives:**

To evaluate sepsis-associated encephalopathy (SAE) research and to quantitatively and qualitatively predict research hot spots using bibliometric analysis.

**Methods:**

We extracted relevant publications from the Web of Science Core Collection on July 28, 2021. We investigated the retrieved data by bibliometric analysis (e.g. co-cited and cluster analysis, keyword co-occurrence) using the software CiteSpace and VOSviewer, the Online Analysis Platform of Literature Metrology (http://bibliometric.com/) and Bibliometrix to analyse and predict the trends and hot spots in this field.

**Main Results:**

We identified 1,582 published articles and reviews on SAE from 2001 to 2021. During this period, the number of manuscripts on SAE increased steadily and peaked in 2021. The USA and China were the leading countries that had a critical impact on SAE research. Among all institutions, Vanderbilt University and Pittsburgh University held leading positions and became central in the collaboration network. Among all the journals, *Critical Care Medicine* published the maximum number of manuscripts in the field of SAE within 20 years. Dal-Pizzol Felipe was the most productive author (61 papers) and received the largest number of citations (930 citations). Co-citation cluster analysis revealed that the most popular terms on SAE in the manner of cluster labels were critical illness, sepsis-associated encephalopathy, polymicrobial sepsis, posterior reversible encephalopathy syndrome, rat brain, intensive care unit, prior sepsis, molecular hydrogen, inflammation drive, metabolic encephalopathies, delirium pathophysiology, and clinical neuroscience. Keyword burst detection indicated that neuroinflammation, blood-brain barrier (BBB) and mitochondria dysfunction were the current research hot spots.

**Conclusions:**

Our study revealed that neuroinflammation, blood-brain barrier, and mitochondria dysfunction had been the research foci of SAE over the past 20 years. These have emerged as the basis for transformation from basic research to clinical application in finding effective methods for the prevention and treatment of SAE.

## Introduction

Sepsis-associated encephalopathy (SAE) is defined as a state of diffuse cerebral dysfunction caused by the inflammatory response of the body to various infections; this inflammatory process does not directly affect the central nervous system (CNS) and presents primarily symptom as a disturbed level of consciousness. The severity of SAE can range from delirium to coma. Disturbance of consciousness is an early warning sign of developing sepsis in a majority of cases (1–5). At present, SAE was reported by some studies to have an incidence of as high as 42% (6), with a higher case fatality rate of 28–180 days, compared with that of non-SAE. SAE is one of the risk factors for death in patients with sepsis (3, 4, 7). When SAE appears as one of the main manifestations of multiple organ dysfunction, the case fatality rate can reach 70% (2). SAE not only increases short-term morbidity and length of stay but may also lead to long-term physical and cognitive impairment. Residual cognitive impairment after surviving sepsis is present in 12.5–21% of patients and includes disorders in attention, processing speed, association learning, visual perception, working memory, and language memory.

Since the 2001s, a substantial quantity of publications on SAE has emerged, based on some pioneering findings in the 1990s that implied better assessment of peri-operative risk factors and relatively safe operations (8–10). However, much less is known about the exact pathogenesis, consistent nomenclature, and diagnostic criteria of SAE, and no study has focused on the analysis or prediction of research hot topics or trends. For these reasons, analysis of research hot spots combined with clinical practice is necessary to improve our understanding of SAE. This article presented a bibliometric review of the articles on SAE based on the data obtained from the Web of Science Core Collection (WoSCC). It sought to identify the publication trends, shifts of hotspots and research fronts, and intellectual milestones in the field of SAE. The results of this work may be helpful in future research planning and decision-making.

## Materials and Methods

### Data Sources and Search Strategies

We conducted a comprehensive literature search within the Web of Science Core Collection (WoSCC) database (Clarivate Analytics, Philadelphia, PA, USA) using the following search strategy: Topic = (Sepsis-Associated Encephalopathy) AND Language = English, with a limited time frame set from 2001 to 2021. We applied filters to limit the search to article and review, index = Science Citation Index Expanded (SCI-EXPANDED), timespan = 2001–2020. We completed all literature retrieval and data downloads in 1 day on July 28, 2021, in order to reduce the bias incurred by frequent database updates.

### Data Collection

Two researchers (Sifan Chen and Yizhe Zhang) independently conducted the primary data search. The manuscripts were screened and recorded for titles, authors, countries, institutions, journals, and the total/ average citation numbers. The WoSCC data were converted to.txt format and imported into the Online Analysis Platform of Biobliometry (http://bibliometric.com/); Bibliometrix (11); CiteSpace V5.7.R5 SE, 64bit (Drexel University, Philadelphia, PA, USA) and VOSviewer 1.6.15 (12) (Leiden University, Leiden, The Netherlands) for further bibliometric analysis.

### Bibliometric Analysis

In our study, we intended to describe all literature characteristics, including countries/institutions, journals, highly cited articles, clustered networks of co-cited references, and keywords with the strongest citation bursts. We obtained the impact factor, category quartile from the Journal Citation Reports 2020 and the PlumX Metrics, which are vital indicators for measuring the scientific value of research. The annual publication numbers, growth tendencies, and citation information of the different countries/ institutions/ authors were analyzed using the online bibliometric platform and R package Bibliometrix. For collaboration analysis, co-citation in combination with cluster analysis, and keyword analysis, we applied CiteSpace and the VOSviewer software to visualize the bibliometric data. To further explore the research front, we searched ClinicalTrials.gov and analyzed the content of the most relevant SAE clinical studies, in accordance with the cluster labels generated by the CiteSpace co-citation analysis of reference, which also represented the forefront research topics. Lastly, we especially extracted the most recent original articles for the last 2 years (2020 and 2021) and analyzed the top-cited articles and highly influential references in the co-citation network to help us catch up with newly emerging concepts.

## Results

### An Overview of Sepsis-Associated Encephalopathy Publications

**Figure 1:**
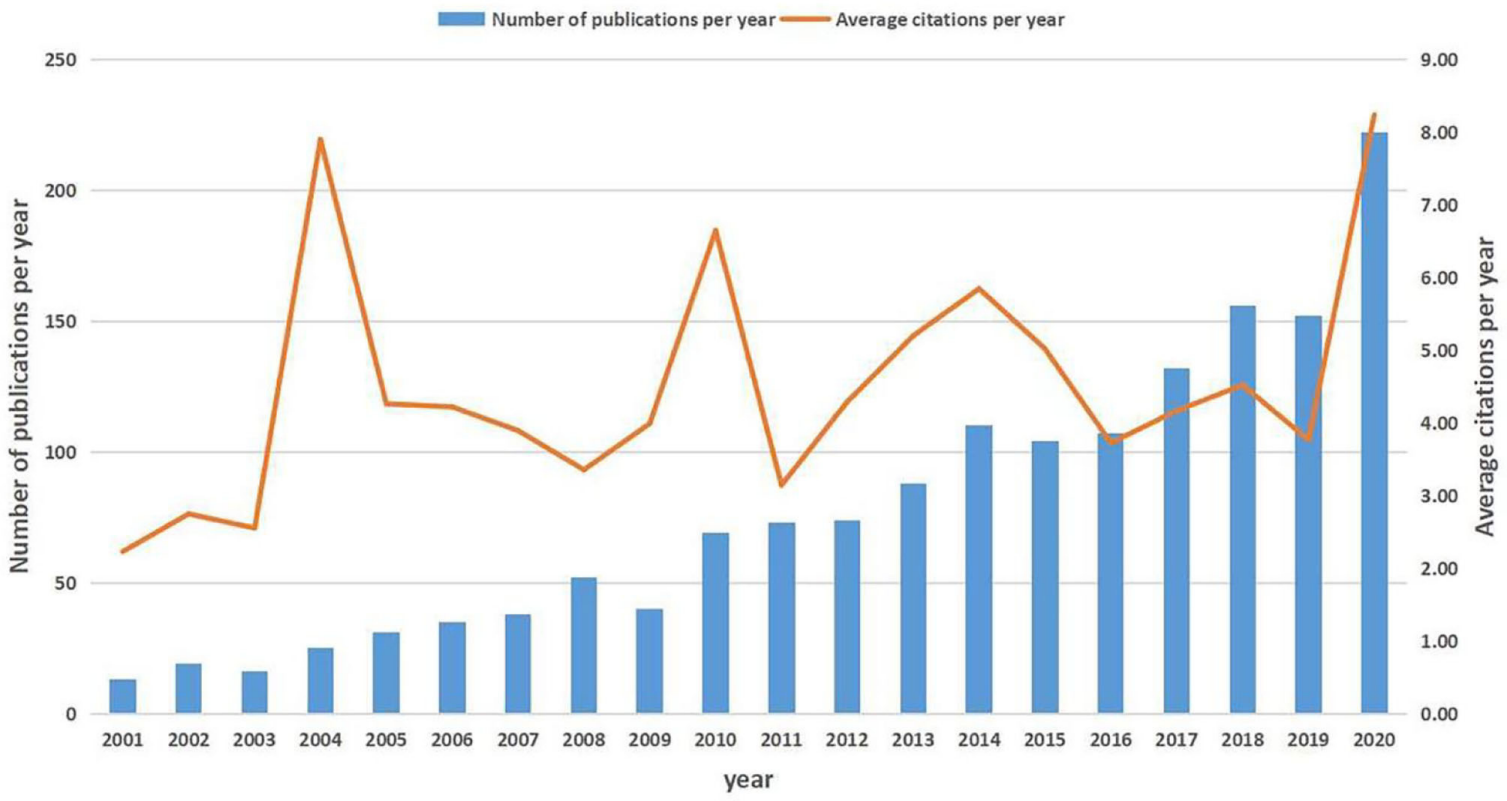
The number of annual publications on SAE research from 2001 to 2020.

After removing unmatched data, a total of 1,582 publications (1,378 articles and 204 reviews) met our inclusion criteria (Supplementary Figure 1A). These publications have been cited 49,337 times in total, and the average number of citations per article was 31.19 times. The overall trend of publication numbers showed a consistent increase from 2001 to 2020, with a peak in 2020. The annual growth rate was 16.11% for scientific production, but the annual citation kept fluctuating in the past 20 years, ranging from 4.00 to 8.00 (Figure 1). Supplementary Table 1 shows the top 25 productive countries that contributed to SAE research. The largest number of documents was published by the USA (*n* = 553, 34.96%), followed by China (*n* = 219, 13.84%). A collaboration network of countries was observed (Supplementary Figure 1B). Of the top 25 most prolific research institutions, 14 were located in the USA, 2 in China, 5 in Brazil, 2 in Canada, 1 in Turkey, and 1 in Germany. The research network among the institutions showed a low-density map (density = 0.0054) (Supplementary Figure 1C). The low-density map of the research network among the institutions meant that the research groups were comparatively dispersed throughout various institutions and that more academic collaborations are needed. In the past 20 years, 654 journals have published articles on SAE research, and the top 10 most active journals are shown in Supplementary Table 2. The highest-ranking journal was Critical Care Medicine, which had a citation number of 719, and the second was Critical Care, which had a citation number of 538. The 1,582 publications were made by 8,230 authors. Supplementary Table 3 shows the top 25 productive authors and their contributions to SAE research between 2001 and 2020. The use of CiteSpace to generate the author collaboration network of SAE research resulted in 657 nodes and 944 links (Supplementary Figure 1D). The knowledge map can provide information on influential scholars or research groups and can help in the search for potential collaborators in the field. The leading author in the field was Dal-Pizzol Felipe from the University of Southern Santa Catarina, Brazil, with 61 publications to his credit, followed by Quevedo Joao from the Univ Southern Santa Catarina UNESC, Brazil (48 publications).

### Keyword Co-occurrence Analysis of Sepsis-Associated Encephalopathy Hot Spots

By analyzing the contents in the titles and abstracts of the included manuscripts, VOSviewer identified 182 author keywords and Keywords Plus with a minimum occurrence of 15 times, visualizing the data with a bubble map. On VOSviewer visualization, the number of occurrences was represented by the size of the bubble and the assigned color identifying the cluster.

**Figure 2:**
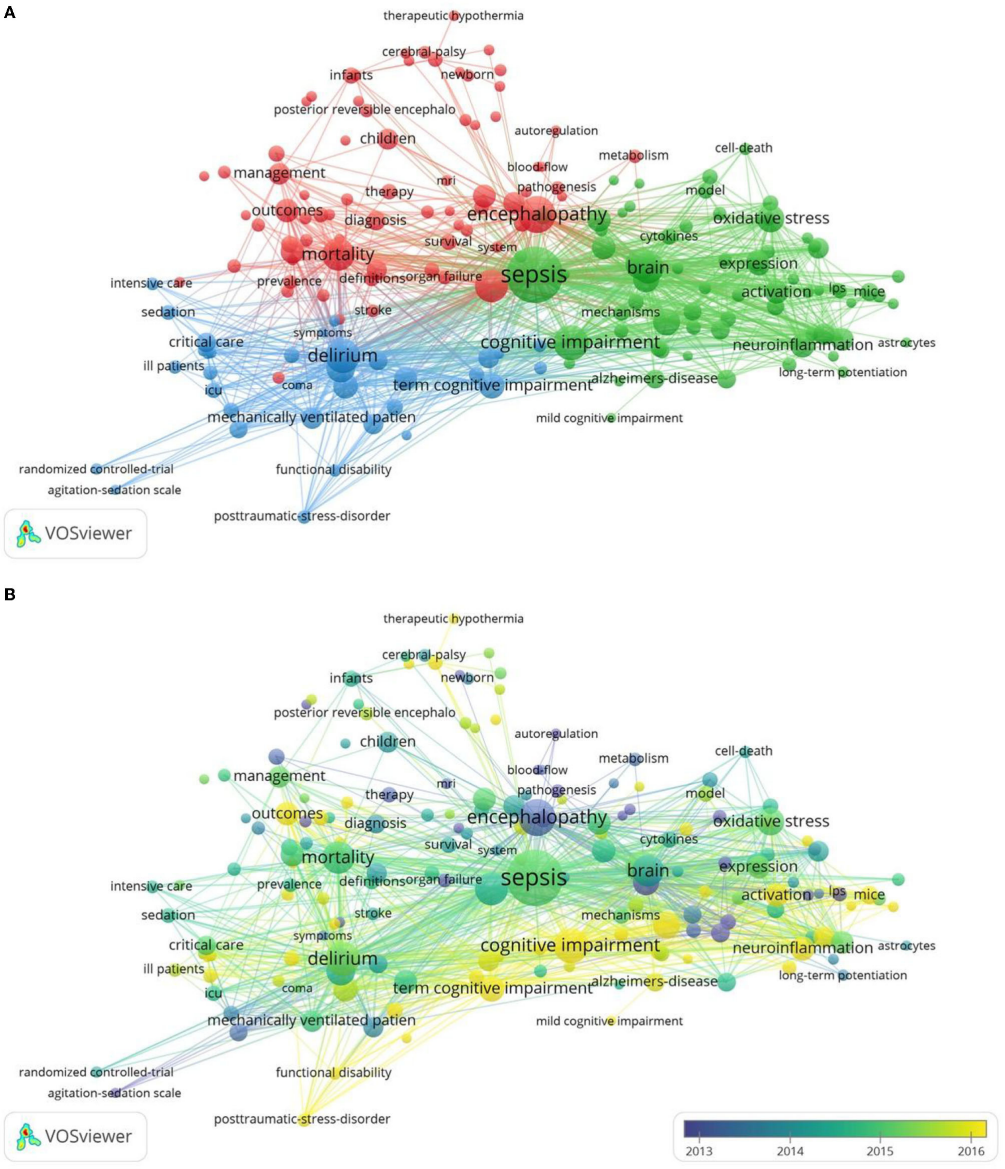
The network map of keyword clustering showed 182 keywords with a minimal occurrence of 15 times and classified into 3 clusters **(A)** and their average publication year **(B)**.

In our analysis, VOSviewer divided the keywords of the SAE field into three major clusters (Figure 2A). This approach was based on the fact that more frequent co-occurrence in the matrix meant stronger the linkage among the keywords. The red cluster focused on the correlation between SAE and patient prognosis and speculates that SAE could be an independent risk factor for survival. On the other hand, the blue cluster focused on the impairment of cognitive function by SAE and its associated factors. The green cluster concentrated on the study of SAE pathogenesis, such as neuroinflammation and oxidative stress. Over time, the onset of sepsis has been established to be associated with cognition, and research attention has gradually shifted to changes in cognitive function and its pathogenesis (Figure 2B).

### Top 10 Cited Articles and Plum X Metrics Analysis

The top 10 cited articles are demonstrated in Table 1. The highest number of citations was E.Wesley Ely in JAMA (1,693 citations), followed by Theodore J. Iwashyna also in JAMA (1,155 citations). These articles were often recognized as fundamental in SAE research. Recently, social media has been gaining popularity and has become integrated into the fabric of academic communication (13). Plum X Metrics of the top 10 cited articles were collected on September 15, 2021 (Table 1). Undoubtedly, the article by E.Wesley Ely in JAMA was the most influential and ranked the first in terms of Citations, Usage, Captures, and Mentions. Timary and Nathalie M. Delzenne in PNAS had the highest scores in social media.

**Table 1:** The top 10 high-cited papers in SAE research during 2001 to 2020.

**Rank**	**Title**	**Journal**	**Corresponding authors**	**Publication year**	**Total citations**	**PlumX metrics**				
						**Citations**	**Usage**	**Captures**	**Mentions**	**Social media**
1	Delirium as a predictor of mortality in mechanically ventilated patients in the intensive care unit	JAMA	E. Wesley Ely	2004	1,693	1,952	2,045	2,025	8	27
2	Long-term cognitive impairment and functional disability among survivors of severe sepsis	JAMA	Theodore J. Iwashyna	2010	1,155	1,223	1,038	1,202	5	70
3	Intestinal permeability, gut-bacterial dysbiosis, and behavioral markers of alcohol-dependence severity	PNAS	Timary & Nathalie M. Delzenne	2014	346	380	205	590	5	74
4	Systemic inflammation induces acute behavioral and cognitive changes and accelerates neurodegenerative disease	BIOLOGICAL PSYCHIATRY	Colm Cunningham	2009	338	344	814	415	/	20
5	Systemic infection and delirium: when cytokines and acetylcholine collide	LANCET	ProfWillem van Gool	2010	297	311	18	338	/	16
6	Posterior reversible encephalopathy syndrome in infection, sepsis, and shock	AMERICAN JOURNAL OF NEURORADIOLOGY	Walter S. Bartynski	2006	292	319	/	/	/	2
7	Population burden of long-term survivorship after severe sepsis in older Americans	JOURNAL OF THE AMERICAN GERIATRICS SOCIETY	Theodore J. Iwashyna	2012	264	257	941	295	1	35
8	Peripheral infection and aging interact to impair hippocampal memory consolidation	NEUROBIOLOGY OF AGING	Ruth M. Barrientos	2012	230	238	247	142	/	/
9	Systemic infection, interleukin 1 beta, and cognitive decline in Alzheimer's disease	JOURNAL OF NEUROLOGY NEUROSURGERY AND PSYCHIATRY	Clive Holmes	2006	227	/	/	/	/	/
10	Continuous electroencephalography in the medical intensive care unit	CRITICAL CARE MEDICINE	Hirsch, Lawrence J.	2009	226	254	4	209	/	3

### Co-citation and Clustered Network Visualization Based on Co-cited References

**Figure 3:**
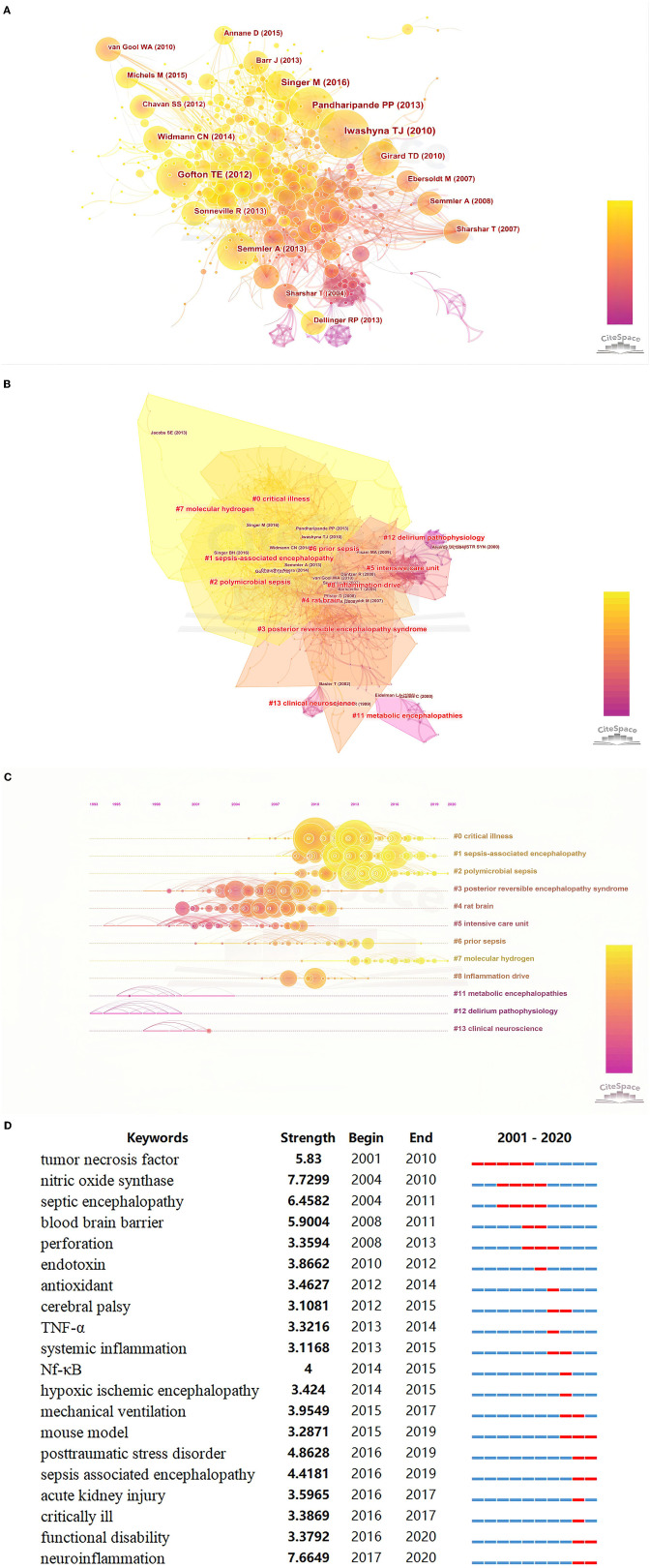
**(A)** Co-citation map of references on SAE; **(B)** The clustered network map of co-cited references on SAE. **(C)** The timeline view of co-citation clusters with their cluster labels on the right. **(D)** Keywords with the strongest citation bursts in original articles on SAE research between 2001 and 2020.

The co-citation and clustered network map were generated by CiteSpace from 52,773 references in a hierarchical order (Figures 3A,B). Visualization of co-cited references showed a total of 886 nodes and 2,446 links (Figure 3A). In this network, each node represented a cited article, and the size of each node was proportional to the total co-citation frequency of the associated article. As shown in Figure 3B, the co-cited references were clustered into 12 major cluster labels including, critical illness, SAE, polymicrobial sepsis, posterior reversible encephalopathy syndrome, rat brain, intensive care unit (ICU), prior sepsis, molecular hydrogen, inflammation drive, metabolic encephalopathies, delirium pathophysiology, and clinical neuroscience. Figure 3C displays the timeline of distinct co-citation and shows that cluster #2 polymicrobial sepsis had the highest degree of citation bursts, and the research foci seemed to transform from posterior reversible encephalopathy syndrome to molecular hydrogen.

A search of ClinicalTrials.gov on July 31, 2021, identified 12 ongoing studies related to SAE. After reading the descriptions of all 12 studies and excluding all irrelevant research, the research themes of the remaining 11 studies were categorized (Table 2). Of the top 11 research themes, 1–3 was consistent with the CiteSpace cluster analysis result inflammation drive. The clinical trial numbered NCT04076826 thought that the pathophysiology of SAE was related to increased levels of inflammatory mediators, such as tumor necrosis factor (TNF)-α, interleukin (IL)-1, and IL-6, which lead to neuronal inflammation. In experimental models of sepsis, DEX has been shown to decrease the production of inflammatory mediators. The clinical trials numbered NCT00772096 and NCT01029080 thought that SAE is caused by an unspecific reaction of the brain to an intense inflammatory stimulus and that the inflammatory response and its effects on the brain can be therapeutically influenced. Of the top 11 research themes, 4–5 were consistent with the CiteSpace cluster analysis result ICU. The clinical trials numbered NCT02288715 and NCT04467762 had a consensus that reducing the incidence of SAE reduced ICU length of stay and significantly improved survival. Of the top 11 research themes, 6–10 were consistent with the CiteSpace cluster analysis result clinical neuroscience. They suggested that cerebral blood flow regulation has an important role in the pathogenesis of SAE and that the development of septic encephalopathy may be predicted by early measurements of cerebral perfusion or EEG. The last research themes were consistent with the CiteSpace cluster analysis result of metabolic encephalopathies. The clinical trial numbered NCT03133208 thought that injured neurons release neuron-specific proteins that disturb metabolic pathways and could have diagnostic relevance in SAE.

**Table 2:** Eleven clinical trials exploring sepsis-associated encephalopathy that were registered with ClinicalTrials.gov.

**NCT number**	**Research theme**	**Status**	**Study title**	**Conditions**	**Sample size (n)**	**Study type**	**Study design**
NCT04076826	Inflammation drive	Recruiting	Adjunctive sedation with dexmedetomidine for the prevention of severe inflammation and septic encephalopathy	Sepsis-Associated Encephalopathy	60	Interventional	Allocation: Randomized|Intervention Model: Parallel Assignment|Masking: None (Open Label)|Primary Purpose: Prevention
NCT00772096	Inflammation drive	Completed	Septic encephalopathy and late cognitive dysfunction	Sepsis|Severe Sepsis	50	Interventional	Allocation: Randomized|Intervention Model: Parallel Assignment|Masking: Triple (Participant, Investigator, Outcomes Assessor)|Primary Purpose: Prevention
NCT01029080	Inflammation drive	Completed	Cerebrovascular autoregulation during sepsis	Sepsis|Delirium	30	Observational	Observational Model: Case-Only|Time Perspective: Prospective
NCT02288715	Intensive care unit	Completed	Relationship of cerebral perfusion pressure variability to sepsis-associated encephalopathy	Sepsis	110	Observational	Observational Model: Case-Control|Time Perspective: Prospective
NCT04467762	Intensive care unit	Recruiting	Neurocognitive impairment in pediatric patients with meningoencephalitis and sepsis-associated encephalopathy	Critical Illness|Brain Injuries| Encephalopathy|Delirium| Inflammatory Disease| Central Nervous System Diseases	30	Observational	Observational Model: Case-Control| Time Perspective: Prospective
NCT04870983	clinical neuroscience	Recruiting	The dynamic change of MMN in patients with sepsis associated encephalopathy	Sepsis-Associated Encephalopathy|Mismatch Negativity	84	Observational	Observational Model: Cohort|Time Perspective: Prospective
NCT00410111	clinical neuroscience	Completed	Pathogenesis and cerebrovascular manifestations of septic encephalopathy	Sepsis	23	Observational	Observational Model: Cohort|Time Perspective: Prospective
NCT03562689	clinical neuroscience	Suspended	Cognitive processing in patients surviving delirium. neuropsychological, EEG and structural brain correlates.	Septic Encephalopathy| Delirium|Sepsis	42	Observational	Observational Model: Cohort|Time Perspective: Retrospective
NCT02442986	clinical neuroscience	Completed	Neurological outcome in surgical and non-surgical septic patients	Critical-Illness| Polyneuropathy|Myopathy| Septic Encephalopathy	32	Observational	Observational Model: Case-Control|Time Perspective: Prospective
NCT03173196	clinical neuroscience	Completed	Prospective analysis of septic associated encephalopathy using the non-invasive acoustocerebrography-ACG	Encephalopathy|Organ Dysfunction Syndrome	20	Observational	Observational Model: Case-Control|Time Perspective: Prospective
NCT03133208	metabolic encephalopathies	Recruiting	Sepsis associated encephalopathy (SAE) biomarkers	Sepsis|Altered Mental Status|Sepsis-Associated Delirium| Sepsis Associated Encephalopathy|Delirium, Sepsis Associated	90	Observational	Observational Model: Cohort|Time Perspective: Prospective

### Burst Detection With Keywords

Burst detection identified the emerging concepts that have drawn the attention of peer investigators. Keywords bursts indicated possible hot research topics. We detected keywords burst between 2001 and 2020 based on the analysis of the 1,582 articles from the WoSCC database. The timeline was depicted as a year-sliced blue line, and the time interval of a burst was marked as a red section on the blue timeline to indicate the beginning/ending year and the duration of a citation burst. We excluded keywords that had little or no research significance and particularly focused on keywords that were representative of the research trends on SAE (Figure 3D). From 2001 to 2020, burst strength was the highest for neuroinflammation (7.6649), followed by septic encephalopathy (6.4582), blood-brain barrier (5.9004), TNF (5.83), post-traumatic stress disorder (4.8628), and SAE (4.4181). Since 2002, the focus was on TNF and CNS, followed by septic encephalopathy, BBB, and perforation. In the subsequent years, some keyword bursts, such as endotoxin, antioxidant and cerebral palsy, continued for only a short period of time. Since 2016, the most recent and strongest keyword burst was that for neuroinflammation and post-traumatic stress disorder.

### Analysis of the Most Recent Publications and Their Co-citation Network

To better clarify the research front in studying SAE, we particularly analyzed the research output in the recent 2 years (2020–2021). We used the same search strategies as described in the methods and limited the document type to the only original article, in order to focus on the original conceptualization and to obtain a deep exploration into the research front. A total of 285 articles were identified in the WoSCC; Table 3 lists the top-cited articles in the past 2 years. We extracted all the literature in a.txt format and imported these into CiteSpace software to construct a co-citation network. The co-citation network modeling opened up a relatively wide range of intellectual base for SAE research and might help identify the high-impact contributions in recent years. In this study, we listed the top 10 most influential references using centrality, which is a commonly used structural metric. Studies have suggested that nodes with high centrality values tend to play a more significant role in the network (14).

**Table 3:** Top cited original articles in 2020 and 2021 (ranked by citation counts).

**Rank**	**Title**	**Journal**	**Corresponding authors**	**Publication year**	**Total citations/** **centrality**
1	Molecular hydrogen attenuates sepsis-induced neuroinflammation through regulation of microglia polarization through an mTOR-autophagy-dependent pathway	INTERNATIONAL IMMUNOPHARMACOLOGY	Yu, Y; Yu, YH	2020	17
2	Mitochondrial dysfunction mediated through dynamin-related protein 1 (Drp1) propagates impairment in blood brain barrier in septic encephalopathy	JOURNAL OF NEUROINFLAMMATION	Haileselassie, B; Joshi, AU	2020	14
3	Fish oil–rich lipid emulsion modulates neuroinflammation and prevents long-term cognitive dysfunction after sepsis	NUTRITION	Petronilho, F	2020	12
4	Plasma miR-370-3P as a Biomarker of Sepsis-Associated Encephalopathy, the Transcriptomic Profiling Analysis of Microrna-Arrays From Mouse Brains	SHOCK	Leelahavanichkul, A	2020	9
5	NADPH oxidase 2 as a potential therapeutic target for protection against cognitive deficits following systemic inflammation in mice	BRAIN BEHAVIOR AND IMMUNITY	Wu, HM	2020	9
6	Hydrogen gas alleviates blood-brain barrier impairment and cognitive dysfunction of septic mice in an Nrf2-dependent pathway	INTERNATIONAL IMMUNOPHARMACOLOGY	Wang, CY; Yu, YH	2020	8
7	Hydrogen attenuates sepsis-associated encephalopathy by NRF2 mediated NLRP3 pathway inactivation	INFLAMMATION RESEARCH	Chen, HG	2020	7
8	Loss of tissue-nonspecific alkaline phosphatase (TNAP) enzyme activity in cerebral microvessels is coupled to persistent neuroinflammation and behavioral deficits in late sepsis	BRAIN BEHAVIOR AND IMMUNITY	Brown, CM	2020	5
9	Mitochondrial Transplantation Attenuates Brain Dysfunction in Sepsis by Driving Microglial M2 Polarization	MOLECULAR NEUROBIOLOGY	Wang, Q	2020	5
10	MicroRNA miR-126 attenuates brain injury in septic rats *via* NF-κB signaling pathway	BIOENGINEERED	Huang, YY	2021	4
11	Infiltrated regulatory T cells and Th2 cells in the brain contribute to attenuation of sepsis-associated encephalopathy and alleviation of mental impairments in mice with polymicrobial sepsis	BRAIN BEHAVIOR AND IMMUNITY	Inoue, S	2021	3
12	Hydrogen Gas Alleviates Sepsis-Induced Brain Injury by Improving Mitochondrial Biogenesis Through the Activation of PGC-alpha in Mice	SHOCK	Xie, KL	2021	3
13	INT-777 prevents cognitive impairment by activating Takeda G protein-coupled receptor 5 (TGR5) and attenuating neuroinflammation *via* cAMP/ PKA/ CREB signaling axis in a rat model of sepsis	EXPERIMENTAL NEUROLOGY	Gong, Y	2021	2
14	Pharmacological and genetic inhibition of translocator protein 18 kDa ameliorated neuroinflammation in murine endotoxemia model	SHOCK	Aizawa, H	2021	1
15	Gold nanoparticles reduce inflammation in cerebral microvessels of mice with sepsis	JOURNAL OF NANOBIOTECHNOLOGY	Rodrigues, SF	2021	1
16	GYY4137 protected the integrity of the blood-brain barrier *via* activation of the Nrf2/ARE pathway in mice with sepsis	FASEB JOURNAL	Cui, W	2021	0
17	Reducing LncRNA-5657 expression inhibits the brain inflammatory reaction in septic rats	NEURAL REGENERATION RESEARCH	Qian, KJ	2021	0
18	Hydrogen gas alleviates sepsis-induced neuroinflammation and cognitive impairment through regulation of DNMT1 and DNMT3a-mediated BDNF promoter IV methylation in mice	INTERNATIONAL IMMUNOPHARMACOLOGY	Yu, Y; Yu, YH	2021	0
19	The Neuroprotective Effect of Short Chain Fatty Acids Against Sepsis-Associated Encephalopathy in Mice	FRONTIERS IN IMMUNOLOGY	Hong, GL; Lu, ZQ	2021	0
20	Artemisinin improves neurocognitive deficits associated with sepsis by activating the AMPK axis in microglia	ACTA PHARMACOLOGICA SINICA	Chen, XH	2021	0
**High-impact references in the co-citation network of SAE research in 2020 and 2021(ranked by publication year)**					
1	The role of microglia activation in the development of sepsis-induced long-term cognitive impairment	BRAIN BEHAVIOR AND IMMUNITY	Dal-Pizzol, F	2015	0.07
2	The third international consensus definitions for sepsis and septic shock (Sepsis-3)	JAMA	Deutschman, CS	2016	0.08
3	Surviving sepsis campaign: international guidelines for management of sepsis and septic shock: 2016	CRITICAL CARE MEDICINE	Rhodes, A	2017	0.08
4	Neurotoxic reactive astrocytes are induced by activated microglia	NATURE	Liddelow, SA	2017	0.11
5	Targeting inflammatory monocytes in sepsis-associated encephalopathy and long-term cognitive impairment	JCI INSIGHT	Kubes, P	2018	0.16
6	Protein kinase C-delta inhibition protects blood-brain barrier from sepsis-induced vascular damage	JOURNAL OF NEUROINFLAMMATION	Kiani, MF	2018	0.02
7	Poldip2 mediates blood-brain barrier disruption in a model of sepsis-associated encephalopathy	JOURNAL OF NEUROINFLAMMATION	Hernandes, MS	2019	0.02
8	Caspase-1 inhibitor exerts brain-protective effects against sepsis-associated encephalopathy and cognitive impairments in a mouse model of sepsis	BRAIN BEHAVIOR AND IMMUNITY	Liu, X	2019	0.06
9	Pro-inflammatory activation of microglia in the brain of patients with sepsis	NEUROPATHOLOGY AND APPLIED NEUROBIOLOGY	Lassmann, H	2019	0.03
10	Mitochondrial dysfunction mediated through dynamin-related protein 1 (Drp1) propagates impairment in blood brain barrier in septic encephalopathy	JOURNAL OF NEUROINFLAMMATION	Haileselassie, B; Joshi, AU	2020	0.02

## Discussion

### Research Trends in Sepsis-Associated Encephalopathy

Based on our qualitative and quantitative survey using the software CiteSpace, VOSviewer, and R package Bibliometrix, scientific output and researchers dedicated to the field of SAE have been increasing continuously over the past two decades. The annual growth rate of 16.11% for scientific production implied a rapid rise in researcher interest in this field. Among these, the study by Ely in (15) proposed for the first time that cognitive dysfunction in patients with sepsis was an independent predictor; this was a precedent for the study on sepsis-related encephalopathy. In 2010, Theodore J Iwashyna (16) proposed that SAE results in a pivotal downturn in patients' ability to live independently. Although the quantity of research had been relatively considerable, integral analysis of research hot spots had been lacking. Our intent was to catalog the attributes of relevant studies and focus on the interpretation of keyword co-occurrence and burst detection to predict and direct future research trends.

In the past two decades, the journals with the most number publications on SAE research were primarily in the research field of anesthesiology and included Critical Care Medicine, Critical Care, Journal of Critical Care, Plos One, Intensive Care Medicine, Molecular Neurobiology, Journal of Neuroinflammation, Brain Research, Shock and Brain Behavior and Immunity. However, as early as 1985, SAE was first recognized and investigated by anesthetists and ICU physicians (17). This indicated that SAE research has become a focus in the field of anesthesiology and intensive care medicine. Analysis of the co-citation reference network and author co-occurrence map (Figure 3A and Supplementary Figure 1D) from 2001 to 2020 indicated that Iwashyna TJ, Pandharipande PP, Gofton TE, Felipe Dalpizzol, and a few other scholars have made impressive achievements that affected the research trends and current understanding of SAE.

### Research Focus on Sepsis-Associated Encephalopathy

Publications that have the highest citation number had been associated with tremendous academic impact on a certain research field. Among the top 10 highly cited publications, we found that the main influenced factors in SAE were neuroinflammation, BBB damage, and microcirculation disturbance. Consistently, Clive Holmes, Barrientos et al. (18) and Bartynski et al. (19) found that basal IL-1β protein levels in the hippocampus did not differ between younger and older rats and that 4 days of lipopolysaccharide (LPS) administration produced substantial elevations in hippocampal IL-1β and caused cognitive impairment in the aged rats but not in adult rats. This was the first proposal on the age-dependent mechanism of SAE. The studies of Leclercq et al. (20) brought forward evidence on the presence of multiple and highly complex pathways underlying the gut-brain axis that involve brain biochemistry, the vagus nerve, proinflammatory cytokines, and tryptophan metabolism. This implied that neuroinflammation remains one of the main pathogenic factors of SAE. On the other hand, cholinergic neurologic imbalance also plays a role in the pathogenesis of SAE. van Gool et al. (21) suggested that the microglial response is usually regulated tightly but that defensive features could make neurotoxic microglial cells escape cholinergic inhibition and may be followed by a self-propelling neuroinflammatory reaction. Based on these considerations, they postulated that impaired cholinergic inhibitory control of microglia in elderly people and, to a greater extent, in patients with (incipient) neurodegenerative disorders contributes to uncontrolled neuroinflammation.

Microcirculation disorder caused by low mean arterial pressure (MAP) is also important in the pathogenesis of SAE. Iwashyna et al. (16) evaluated in a prospective cohort study the cognitive function of patients hospitalized for severe sepsis from 1998 to 2005. Results showed that the mean age of survivors upon hospitalization was 76.9 years. On multivariate regression, the prevalence of moderate to severe cognitive impairment increased by 10.6 percentage points among patients who survived severe sepsis, with an odds ratio of 3.34 and 95% CI of 1.53–7.25. That research demonstrated for the first time that severe sepsis was independently associated with enduring cognitive and functional limitations. Severe sepsis was independently associated with three times the odds of moderate to severe cognitive impairment. Low MAP may directly contribute to brain injury and subsequent cognitive impairment. Oddo et al. (22) suggested that Patients with sepsis had a higher rate of ESZs or PEDs than those without sepsis (32 vs. 9%, *p* < 0.001).

The BBB is important for the maintenance of brain homeostasis. During sepsis, proinflammatory cytokines, such as IL-1β/TNF-α, are responsible for structural alterations in the BBB. Increased permeability of the BBB can lead to the activation of glial cells, such as microglia, and the production of cytotoxic mediators, which in turn act on and further damage the BBB. Deutschman and Tracey (23) indicated that HMGB1 exposure impairs the endothelial barrier function and results in decreased expression of zonula occludens and occludin, which are cytoplasmic membrane proteins that form intercellular tight junctions; this leads to increased BBB permeability. This leak can be large enough to enable the entry of monocytes, lymphocytes, and neutrophils and induce neuroinflammation. Based on this article, the academic community's research on SAE is more focused on the BBB.

### Research Fronts in Sepsis-Associated Encephalopathy

Research fronts are the emerging theories or research topics. Co-cited references provide important information regarding intellectual connections among various scientific concepts. According to the theory of de Solla Price (24), the pattern of bibliographic references indicates the nature of the scientific research front. The high-frequency topics, including SAE, inflammation, and posterior reversible encephalopathy syndrome, in the co-cited reference cluster analysis indicated that inflammation remained in the research front of SAE research. Our evaluation of the top keywords using burst detection showed that BBB and neuroinflammation attracted most of the attention of peer researchers in the past 20 years. Therefore, in the following discussion, we will particularly focus on emerging topic terms and the related publications, which provided valuable insights into the current stage of the research front.

Sepsis-associated encephalopathy is commonly complicated by septic conditions and is responsible for the increased mortality and poor outcomes of patients with sepsis. Although multiple factors, such as inflammatory cytokines, the collapse of the BBB, ischaemic processes, alterations in neurotransmitters, and mitochondrial dysfunction, were reportedly involved in the pathogenesis of SAE, the specific mechanism has not been established. Ren et al. and Dal-Pizzol et al. (25, 26) thought that inhibition of the microglia is beneficial for abating brain oxidative damage and inflammation in sepsis, along with improvements in long-term cognitive function (27). Moreover, astrocytes present with aberrant responses that promote intractable neuroinflammation and cognitive impairment in sepsis (7, 28). Activated astrocytes are capable of releasing multiple inflammatory mediators, such as TNF-α, IL-1β, and IL-6, and further facilitate the development of neuroinflammation (29, 30). Neutrophils release inflammatory cytokines, increase the activity of myeloperoxidase and promote oxidative damage (31–34).

Sepsis-associated encephalopathy is characterized by diffuse brain dysfunction secondary to infection elsewhere in the body without overt CNS infection (35, 36). Perhaps, a change in the permeability of the BBB is an important link in mediating the pathogenesis of SAE. BBB disruption allows the influx of neurotoxic blood-derived debris, cells, and microbial pathogens into the brain and is associated with inflammatory and immune responses, (37–39). Hu et al. (40), Yadav et al. (41), and Cyr et al. (42) indicate that in the early phase of sepsis, nitric oxide exhibits proinflammatory characteristics and contributes to the activation and dysfunction of cerebrovascular endothelial cells. MMP-9 is a type IV collagenase (43). The corresponding natural inhibitor of MMP is TIMP. Under physiologic conditions, the brain structurally expresses MT1-MMP, with low-level expression of MMP-9 and high-level expression of the structural MMP inhibitors TIMP-2 and TIMP-3. This mechanism may play a role in maintaining the importance of the functional integrity of the BBB. However, the activity of MMP was found to significantly increase when the body was hit by a lethal amount of LPS (44). Therefore, in severe sepsis, BBB dysfunction is related to the activities of MMP-2 and MMP-9. Inflammatory factors and oxidative stress increase the expression of MMP, which can also activate inflammatory factors and ultimately strengthen the damaging effect on the BBB (45). In summary, several clinical practices have shown that TIMP, the corresponding natural inhibitor of MMP, is a better choice for perioperative patients.

A large number of studies have found that serum and brain amino acid profiles change during sepsis and suggested that abnormal amino acid profiles may play an important role in SAE (46, 47). Moreover, mitochondrial dysfunction plays a very important role in the pathogenesis of SAE (48, 49). d'Avila et al. (50) demonstrated that uncoupling of oxidative phosphorylation takes place in the brain of septic mice and can compromise tissue bioenergetic efficiency.

Exploration of the pathogenesis of SAE among the top 20 highly cited publications in the recent 2 years persistently revealed neuroinflammation, BBB damage, and microcirculation disturbance. However, the main influencing factors focused on mitochondria, hydrogen molecules, and non-coding RNAs (ncRNAs). The role of mitochondrial health as a mechanistic link between inflammation and cellular damage in neuroinflammatory and neurodegenerative diseases has become increasingly recognized. Haileselassie et al. (51) proved that LPS-injected dysfunction of the BBB was dependent on Drp1-Fis1-mediated mitochondrial dysfunction. Yan et al. (52) found that mitochondrial transplantation may modulate microglial polarization and suppress proinflammatory cytokine secretion, thereby improving long-term cognitive impairment after sepsis. Giga et al. (53) found that ONO-2952 pretreatment suppressed the LPS-induced activation of TSPO-expressing microglia in the hippocampus of mice. Huang et al. (54) suggested that NADPH oxidase 2 (NOX2) contributes to glial activation, with subsequent reduction in BDNF expression, synaptic dysfunction, and cognitive deficits after LPS injection.

Chinese scholars have focused their goals on the exploration of the role of hydrogen molecules in the pathogenesis of SAE. They suggested that the action of hydrogen molecules through the Nrf2/ARE pathway is important in SAE (55–58). GYY4137 is a new synthetic compound that can release lower concentrations of H_2_S in the form of an aqueous solution over a relatively long time period. Cui et al. (55) indicated that GYY4137 exerts a protective role in SAE through the Nrf2/ARE pathway by reducing oxidative stress and inhibiting inflammation and apoptosis. Yu et al. (56) concluded that inhalation of 2% hydrogen gas protected the BBB by decreasing its permeability, thereby, reducing SAE and improving cognitive function, which is mediated through Nrf2 and its downstream signaling pathways. In addition, Zhuang et al. (59) found that molecular hydrogen could attenuate sepsis-induced neuroinflammation by modulating microglia polarization. Yu et al. (60) demonstrated that molecular hydrogen may be a promising agent for alleviating SAE and that 2% H2 inhalation can protect against sepsis-induced neuroinflammation by decreasing DNMT1- and DNMT3a-mediated BDNF promoter IV methylation, enhancing BDNF levels and improving the cognitive dysfunction in septic mice.

Research has demonstrated that ncRNAs are key in the manifestation of diseases at the epigenetic level. In terms of sepsis, several miRNAs have been regarded as biomarkers or prognostic indicators. Nong et al. (61) found that miR-126 expression was decreased in the brain tissue of septic rats. Overexpression of miR-126 can improve brain injury in CLP rats through inhibitions of the NF-κB signaling pathway activity, inflammatory response, and oxidative brain stress. Similarly, Visitchanakun et al. (62) found that miR-370-3p increased in SAE mouse brains after CLP 24 h. Zhan et al. (63) demonstrated that Sh-LncRNA-5657 transfection decreased the expression of LncRNA-5657 in LPS-treated glial cells and decreased the mRNA and protein levels of TNF-α, IL-1β, and IL-6; these results suggested that LncRNA-5657 expression can significantly reduce the inflammatory reaction in SAE and induce protective effects against this condition.

Inflammation and oxidative stress are key players in SAE pathophysiology. Gold nanoparticles have been progressively demonstrated to have important anti-inflammatory properties (64). Di Bella et al. (65) thought that 20-nm cit-AuNP treatment reduced leukocyte and platelet adhesion to cerebral blood vessels prevented BBB failure, reduced TNF-α concentration in the brain, and reduced ICAM-1 expression in both the circulating polymorphonuclear leukocytes and cerebral blood vessels of mice with sepsis.

In addition, fish oil (66) and tissue-non-specific alkaline phosphatase (67) may mediate SAE by affecting the BBB. Moreover, short chain fatty acids (68), Treg/Th2 cells (69), Takeda G protein-coupled receptor 5 (70), and artemisinin (71) can regulate neuroinflammation through immune-mediated microglia activation.

Among the top 10 highly co-cited references in the past 5 years, we found that the main factors that influenced SAE were related to the perfusion pressure to the BBB and neuroinflammation. The Third International Consensus Definitions for Sepsis and Septic Shock that was published in JAMA in 2016 (72) and the International Guidelines for Management of Sepsis and Septic Shock in 2017 (73) both emphasized that patients with septic shock and persistent hypotension that requires vasopressors to maintain a MAP of ≥65 mm Hg, which can avoid sustained brain hypoperfusion that leads to impaired cognitive function. BBB permeability is another global focus on SAE. Tang et al. (74) thought that PKCδ activation altered the structural and functional integrity of the BBB and led to vascular damage and inflammation-induced tissue damage. The PKCδ-TAT peptide inhibitor has therapeutic potential for the prevention or reduction of cerebrovascular injury in sepsis-induced vascular damage. Kikuchi DS (75) found that Poldip2 could mediate LPS-induced BBB destruction by regulating NF-κB/p65 activation and Cox-2 and PGE2. In CLP-induced experimental sepsis, Xu et al. (76) demonstrated that caspase-1 inhibition dramatically down-regulated pyroptosis, reduced inflammatory cytokines release, protected brain ultrastructure, and preserved cognitive functions. Recent studies suggested that LPS-induced BBB injury appeared to be dependent on Drp1/Fis1-mediated mitochondrial dysfunction (51). The neuroinflammatory mechanism in SAE has endured. In 2015, Michels et al. (27) suggested that treatment with minocycline prevented an increase in the markers of oxidative damage and inflammation in the hippocampus after sepsis and was associated with improvement in SAE. In 2017, Liddelow et al. (77) proposed that activated microglia induced A1 astrocytes by secreting IL-1α, TNF, and C1q. Death of atomised CNS neurons *in vivo* can be prevented by blocking the formation of A1 astrocytes. In advance, research on SAE has focused mainly on microglia. Andonegui et al. (31) and Zrzavy et al. (78) found that sepsis contributed to microglia activation and that there was additional recruitment of perivascular macrophages. Proinflammatory microglia activation occurred in the presence of homeostatic microglia cells. In contrast to inflammatory or ischaemic diseases of the brain, acute sepsis does not present with the expression of the anti-inflammatory microglia markers CD163 or CD206.

### Strengths and Limitations

Compared with traditional reviews, analysis based on bibliometric tools, such as CiteSpace, VOSviewer, and Bibliometrix, provide better insight into the evolving research foci and trends and comparatively comprehensive and objective data analysis. However, this study design came with certain limitations. According to our inclusion criteria, only English documents were enrolled; therefore, some important non-English documents might have been excluded. In addition, we only the documents indexed in the WoSCC database because of the limitations of the software CiteSpace. Although most of the research manuscripts on SAE were indexed in the WoSCC database, some other databases, such as Pubmed and Scopus, might ensure a full representation of all available academic outputs in this field.

## Conclusions

We firmly believed that our understanding of SAE has advanced markedly over the past 20 years. With the help of bibliometric mapping, we were able to describe the overall structure of the scientific research on SAE and provided vivid and gathered information to other researchers. The current research focused on neuroinflammation, BBB, and mitochondrial dysfunction, which are critical for the improvement of interventions and prognosis of patients with SAE.

## Data Availability Statement

The original contributions presented in the study are included in the article/Supplementary Material, further inquiries can be directed to the corresponding author.

## Author Contributions

YZ and DS: study conception. YZ, SC, and WT: study design. YZ, HZ, WL, WD, XZ, and XG: study conduct. SC: Data analysis. YZ and SC: had full access to all the data in the study, take responsibility for the integrity of the data and the accuracy of the data analysis, data interpretation, and drafting of the manuscript. YZ, SC, WT, HZ, WL, WD, XZ, XG, and DS: critical revision of the manuscript for important intellectual content. All authors contributed to the article and approved the submitted version.

## Funding

This study was supported by grants from the National Natural Science Foundation of China (Nos. 81771133, 81970995), Shanghai Shenkang Hospital Development Center Founding (SHDC12017X11), Shanghai Municipal Science and Technology Commission Founding (21S31900100), Renji Hospital Clinical Innovation Foundation (PYII20-09), and Shanghai Municipal Education Commission-Gaofeng Clinical Medicine Support [20171916, 20191903]. Shanghai Municipal Commission of Health and Family Planning Funding (201840241). The funders had no role in the analyses and interpretation of the results or writing of the manuscript.

## Conflict of Interest

The authors declare that the research was conducted in the absence of any commercial or financial relationships that could be construed as a potential conflict of interest.

## Publisher's Note

All claims expressed in this article are solely those of the authors and do not necessarily represent those of their affiliated organizations, or those of the publisher, the editors and the reviewers. Any product that may be evaluated in this article, or claim that may be made by its manufacturer, is not guaranteed or endorsed by the publisher.

## Supplementary Material

Supplementary Figure 1 Flow diagram of the included papers **(A)**, the collaboration between countries/regions **(B)**, the network map of institutions involved in SAE research **(C)**, and the network map of productive authors **(D)**.

Supplementary Table 1 The top 25 countries/regions and institutions contributing to publications in SAE research.

Supplementary Table 2 The top 10 most active journals that published articles in SAE research (sorted by count).

Supplementary Table 3 The top 25 most productive authors contributed to publications in SAE research.
