# Supplementary figures and images for: Emerging Trends and Hot Spots in Sepsis-Associated Encephalopathy Research From 2001 to 2021: A Bibliometric Analysis
Source: Front Med (Lausanne). 2022 Feb 28;9:817351. doi: 10.3389/fmed.2022.817351 (PMC8918530; doi:10.3389/fmed.2022.817351)

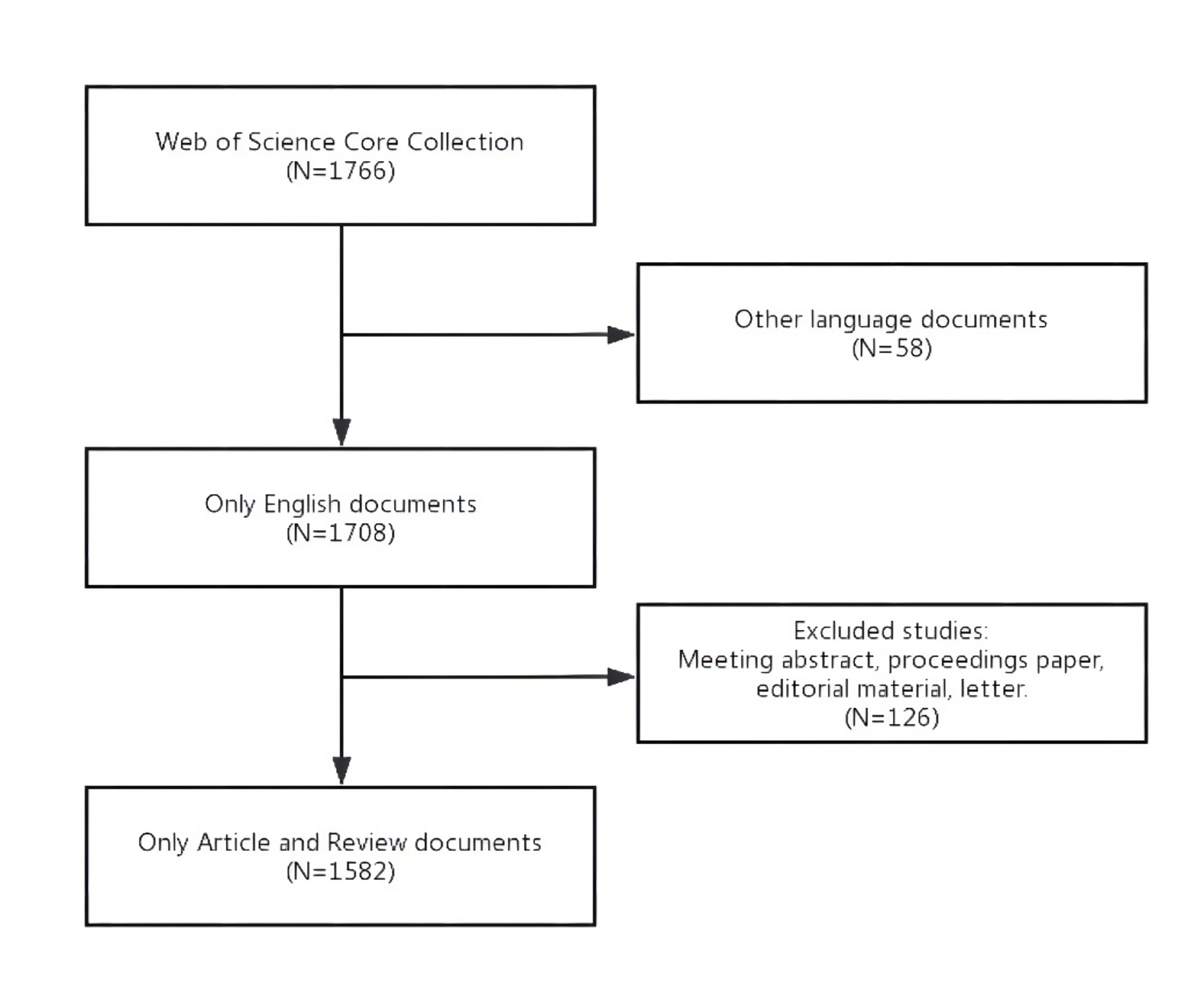

Supplement: Supplementary Figure 1 — Flow diagram of the included papers (A), the collaboration between countries/regions (B), the network map of institutions involved in SAE research (C), and the network map of productive authors (D). [file Data_Sheet_1.ZIP › supplementary figure/Supplementary 1A.jpg]
