# Supplementary material for: Emerging Trends and Hot Spots in Sepsis-Associated Encephalopathy Research From 2001 to 2021: A Bibliometric Analysis
Source: Front Med (Lausanne). 2022 Feb 28;9:817351. doi: 10.3389/fmed.2022.817351 (PMC8918530; doi:10.3389/fmed.2022.817351)
Supplement: Supplementary Table 1 — The top 25 countries/regions and institutions contributing to publications in SAE research. [file Data_Sheet_2.ZIP › supplementary table/supplementary table 1.docx]

| **Supplementary Table 1** The top 25 countries/regions and institutions contributing to publications in SAE research | | | | | | | | | | |  | |  | |
| --- | --- | --- | --- | --- | --- | --- | --- | --- | --- | --- | --- | --- | --- | --- |
| Rank | Country/  region | Article counts | Total  citations |  | Institutions | Article counts | Total number of citations | Average number of citations | Total number of first authors | Total number of first author citations | Average number of first author citations | Country | |  |
| 1 | USA | 553 | 19091 |  | Vanderbilt Univ | 295 | 2816 | 9.55 | 45 | 407 | 9.04 | USA | |  |
| 2 | PEOPLES R CHINA | 219 | 4590 |  | Univ Pittsburgh | 72 | 286 | 3.97 | 17 | 80 | 4.71 | USA | |  |
| 3 | BRAZIL | 134 | 3002 |  | Johns Hopkins Univ | 70 | 298 | 4.26 | 12 | 32 | 2.67 | USA | |  |
| 4 | GERMANY | 105 | 2743 |  | Univ Michigan | 62 | 905 | 14.6 | 17 | 358 | 21.06 | USA | |  |
| 5 | ENGLAND | 101 | 1973 |  | Tennessee Valley Healthcare Syst | 55 | 703 | 12.78 | 0 | 0 | 0 | USA | |  |
| 6 | CANADA | 81 | 1797 |  | Univ Southern Santa Catarina | 54 | 622 | 11.52 | 13 | 173 | 13.31 | Brazil | |  |
| 7 | JAPAN | 75 | 1060 |  | Univ Penn | 53 | 152 | 2.87 | 15 | 34 | 2.27 | USA | |  |
| 8 | FRANCE | 70 | 3146 |  | Univ Toronto | 53 | 41 | 0.77 | 5 | 2 | 0.4 | Canada | |  |
| 9 | ITALY | 63 | 1143 |  | Univ Sao Paulo | 39 | 241 | 6.18 | 16 | 96 | 6 | Brazil | |  |
| 10 | AUSTRALIA | 51 | 1116 |  | Univ Extremo Sul Catarinense | 38 | 753 | 19.82 | 13 | 330 | 25.38 | Brazil | |  |
| 11 | TURKEY | 46 | 614 |  | Istanbul Univ | 38 | 156 | 4.11 | 12 | 28 | 2.33 | Turkey | |  |
| 12 | BELGIUM | 42 | 1305 |  | Univ Washington | 37 | 87 | 2.35 | 12 | 38 | 3.17 | USA | |  |
| 13 | INDIA | 41 | 470 |  | Northwestern Univ | 35 | 45 | 1.29 | 12 | 8 | 0.67 | USA | |  |
| 14 | NETHERLANDS | 40 | 1231 |  | Univ Extremo Catarinense | 31 | 623 | 20.1 | 10 | 225 | 22.5 | Brazil | |  |
| 15 | SWITZERLAND | 40 | 516 |  | Columbia Univ | 31 | 240 | 7.74 | 8 | 50 | 6.25 | USA | |  |
| 16 | SPAIN | 38 | 648 |  | Univ Calgary | 31 | 180 | 5.81 | 5 | 17 | 3.4 | Canada | |  |
| 17 | TAIWAN | 34 | 718 |  | Yale Univ | 30 | 266 | 8.87 | 5 | 32 | 6.4 | USA | |  |
| 18 | IRELAND | 28 | 1624 |  | Jena Univ Hosp | 30 | 53 | 1.77 | 5 | 10 | 2 | Germany | |  |
| 19 | DENMARK | 25 | 548 |  | Sichuan Univ | 30 | 49 | 1.63 | 11 | 29 | 2.64 | China | |  |
| 20 | SOUTH KOREA | 24 | 212 |  | Univ Texas Hlth Sci Ctr Houston | 29 | 175 | 6.03 | 4 | 10 | 2.5 | USA | |  |
| 21 | ISRAEL | 22 | 380 |  | Zhejiang Univ | 29 | 70 | 2.41 | 13 | 32 | 2.46 | China | |  |
| 22 | SWEDEN | 19 | 42 |  | Mayo Clin | 27 | 95 | 3.52 | 5 | 32 | 6.4 | USA | |  |
| 23 | PORTUGAL | 17 | 418 |  | Univ Utah | 26 | 33 | 1.27 | 4 | 1 | 0.25 | USA | |  |
| 24 | EGYPT | 14 | 55 |  | Univ Fed Rio Grande do Sul | 25 | 194 | 7.76 | 4 | 15 | 3.75 | Brazil | |  |
| 25 | AUSTRIA | 13 | 115 |  | Univ Calif San Francisco | 25 | 53 | 2.12 | 3 | 0 | 0 | USA | |  |
