# Supplementary material for: Emerging Trends and Hot Spots in Sepsis-Associated Encephalopathy Research From 2001 to 2021: A Bibliometric Analysis
Source: Front Med (Lausanne). 2022 Feb 28;9:817351. doi: 10.3389/fmed.2022.817351 (PMC8918530; doi:10.3389/fmed.2022.817351)
Supplement: Supplementary Table 1 — The top 25 countries/regions and institutions contributing to publications in SAE research. [file Data_Sheet_2.ZIP › supplementary table/supplementary table 2.docx]

| **Supplementary Table 2** The top 10 most active journals that published articles in SAE research (sorted by count) | | | | | | |
| --- | --- | --- | --- | --- | --- | --- |
| Rank | Journal title | Country | Quartile  in category (2020) | Article counts | Total number of citations | Average number of citations |
| 1 | CRITICAL CARE MEDICINE | USA | Q1 | 57 | 719 | 12.61 |
| 2 | CRITICAL CARE | UK | Q1 | 48 | 538 | 11.21 |
| 3 | JOURNAL OF CRITICAL CARE | USA | Q3 | 30 | 65 | 2.17 |
| 4 | PLOS ONE | USA | / | 26 | 82 | 3.15 |
| 5 | INTENSIVE CARE MEDICINE | USA | Q1 | 24 | 403 | 16.79 |
| 6 | MOLECULAR NEUROBIOLOGY | USA | Q1 | 24 | 223 | 9.29 |
| 7 | JOURNAL OF NEUROINFLAMMATION | UK | Q1 | 21 | 194 | 9.24 |
| 8 | BRAIN RESEARCH | USA | Q3 | 16 | 52 | 3.25 |
| 9 | SHOCK | USA | Q2 | 14 | 89 | 6.36 |
| 10 | BRAIN BEHAVIOR AND IMMUNITY | USA | Q1 | 13 | 131 | 10.08 |
