# Supplementary material for: Emerging Trends and Hot Spots in Sepsis-Associated Encephalopathy Research From 2001 to 2021: A Bibliometric Analysis
Source: Front Med (Lausanne). 2022 Feb 28;9:817351. doi: 10.3389/fmed.2022.817351 (PMC8918530; doi:10.3389/fmed.2022.817351)
Supplement: Supplementary Table 1 — The top 25 countries/regions and institutions contributing to publications in SAE research. [file Data_Sheet_2.ZIP › supplementary table/supplementary table 3.docx]

**Supplementary Table 3** The top 25 most productive authors contributed to publications in SAE research

**able 3** The top 10 most productive authors and co-cited authors contributed to publications in POCD research

**Table 3** The top 10 most productive authors and co-cited authors contributed to publications in POCD research

| Rank | Author | Article counts | Total number of citations | Average number of citations | First author counts | First author citations counts | Average first author citation counts | Corresponding author | Corresponding author citation counts |
| --- | --- | --- | --- | --- | --- | --- | --- | --- | --- |
| 1 | Dal-Pizzol, F | 61 | 930 | 15.25 | 2 | 48 | 24 | 27 | 609 |
| 2 | Quevedo, J | 48 | 839 | 17.48 | 0 | 0 | 0 | 9 | 120 |
| 3 | Ely, EW | 44 | 708 | 16.09 | 4 | 129 | 32.25 | 5 | 136 |
| 4 | Barichello, T | 39 | 545 | 13.97 | 7 | 242 | 34.57 | 3 | 17 |
| 5 | Petronilho, F | 32 | 462 | 14.44 | 2 | 19 | 9.5 | 15 | 112 |
| 6 | Girard, TD | 30 | 280 | 9.33 | 5 | 105 | 21 | 7 | 115 |
| 7 | Jackson, JC | 29 | 249 | 8.59 | 3 | 31 | 10.33 | 3 | 31 |
| 8 | Comim, CM | 28 | 435 | 15.54 | 13 | 194 | 14.92 | 5 | 32 |
| 9 | Pandharipande, PP | 26 | 242 | 9.31 | 1 | 26 | 26 | 3 | 16 |
| 10 | Sharshar, T | 25 | 622 | 24.88 | 4 | 209 | 52.25 | 11 | 232 |
| 11 | Yang, JJ | 21 | 101 | 4.81 | 1 | 0 | 0 | 10 | 23 |
| 12 | Ji, MH | 19 | 99 | 5.21 | 6 | 27 | 4.5 | 6 | 33 |
| 13 | Thompson, JL | 19 | 225 | 11.84 | 0 | 0 | 0 | 0 | 0 |
| 14 | Michels, M | 18 | 179 | 9.94 | 7 | 115 | 16.43 | 0 | 0 |
| 15 | Annane, D | 15 | 475 | 31.67 | 0 | 0 | 0 | 4 | 200 |
| 16 | Bozza, FA | 14 | 173 | 12.36 | 2 | 66 | 33 | 4 | 75 |
| 17 | Ritter, C | 14 | 202 | 14.43 | 2 | 11 | 5.5 | 2 | 10 |
| 18 | Bozza, FA | 14 | 173 | 12.36 | 2 | 66 | 33 | 4 | 75 |
| 19 | Ritter, C | 14 | 202 | 14.43 | 2 | 11 | 5.5 | 2 | 10 |
| 20 | Danielski, LG | 13 | 93 | 7.15 | 3 | 36 | 12 | 0 | 0 |
| 21 | Streck, EL | 13 | 217 | 16.69 | 1 | 31 | 31 | 2 | 27 |
| 22 | Iwashyna, TJ | 13 | 351 | 27 | 2 | 293 | 146.5 | 2 | 293 |
| 23 | Vincent, JL | 13 | 129 | 9.92 | 0 | 0 | 0 | 3 | 39 |
| 24 | Bernard, GR | 13 | 277 | 21.31 | 0 | 0 | 0 | 0 | 0 |
| 25 | Danielski, LG | 13 | 93 | 7.15 | 3 | 36 | 12 | 0 | 0 |
